# Targeting type 2 inflammation in bullous pemphigoid: current and emerging therapeutic approaches

**DOI:** 10.3389/fmed.2023.1196946

**Published:** 2023-08-08

**Authors:** Wu Han Toh, Hua-En Lee, Chun-Bing Chen

**Affiliations:** ^1^Institute for NanoBioTechnology, Johns Hopkins University, Baltimore, MD, United States; ^2^Translational Tissue Engineering Center, Johns Hopkins University School of Medicine, Baltimore, MD, United States; ^3^Department of Computer Science, Johns Hopkins University, Baltimore, MD, United States; ^4^Department of Biology, Johns Hopkins University, Baltimore, MD, United States; ^5^Cancer Vaccine and Immune Cell Therapy Core Laboratory, Chang Gung Memorial Hospital, Chang Gung Immunology Consortium, Taoyuan, Taiwan; ^6^Department of Dermatology and Drug Hypersensitivity Clinical and Research Center, Chang Gung Memorial Hospital, Taipei, Taiwan; ^7^College of Medicine, Chang Gung University, Taoyuan, Taiwan; ^8^Chang Gung Immunology Consortium, Chang Gung Memorial Hospital, Chang Gung University, Taoyuan, Taiwan; ^9^Department of Dermatology, Xiamen Chang Gung Hospital, Xiamen, China; ^10^Xiamen Chang Gung Allergology Consortium, Xiamen Chang Gung Hospital, Xiamen, China

**Keywords:** bullous pemphigoid, anti-BP180 IgE, eosinophil, type 2 inflammation, rituximab, omalizumab, dupilumab, clinical trials

## Abstract

Bullous pemphigoid (BP) is one of the most common autoimmune bullous diseases and mainly affects an elderly population with multi-morbidity. Due to the frailty of many BP patients, existing treatment options are limited. The blisters associated with BP result from IgG and IgE autoantibodies binding to the central components of hemidesmosome, BP180, and BP230, stimulating a destructive inflammatory process. The known characteristic features of BP, such as intense pruritus, urticarial prodrome, peripheral eosinophilia, elevated IgE, as well as recent expanding evidence from *in vitro* and *in vivo* studies implicate type 2 inflammation as an important driver of BP pathogenesis. Type 2 inflammation is an inflammatory pathway involving a subset of CD4+ T cells that secrete IL-4, IL-5, and IL-13, IgE-secreting B cells, and granulocytes, such as eosinophils, mast cells, and basophils. It is believed that effectors in type 2 inflammation may serve as novel and effective treatment targets for BP. This review focuses on recent understandings of BP pathogenesis with a particular emphasis on the role of type 2 inflammation. We summarize current clinical evidence of using rituximab (B-cell depletion), omalizumab (anti-IgE antibody), and dupilumab (anti-IL-4/13 antibody) in the treatment of BP. The latest advances in emerging targeted therapeutic approaches for BP treatment are also discussed.

## Introduction

Bullous pemphigoid (BP) is one of the most common autoimmune bullous diseases and is associated with tissue-bound and circulating autoantibodies directed against BP antigen 180 (BP180, BPAG2, or type XVII collagen) and BP antigen 230 (BP230 or BPAG1e—epithelial isoform) (1). The two autoantigens are both components of the hemidesmosome, a multiprotein complex of the dermal–epidermal junction providing adhesion between basal keratinocytes and dermal extracellular matrix (2). Clinically, BP is characterized by severe pruritus and tense bullae forming over urticarial plaques. Before blister formation, the disease is typically preceded for several weeks and even months by a prodromal phase in which pruritus alone or in association with excoriated, eczematous, papular, or urticarial lesions occur (3). More than 20% of BP patients may not present with typical blistering. Consequently, these patients often have a long diagnostic delay (4, 5).

BP mainly affects the elderly, with onset usually in the late 70s (3). There is a significant association of BP with neurologic diseases, especially stroke, dementia, Parkinson's disease, and multiple sclerosis (6, 7). Moreover, in a recent case–control study, 84% of subjects with BP had more than two chronic diseases as compared to 65% in controls (8). BP's association with old age and multimorbidity results in limited treatment options and more complications associated with disease and treatment.

In particular, there has been a remarkable trend of increased incidence of BP, a 1.9- to 4.3-fold rise, over the past two decades. Possible explanations for this trend include increasing life expectancy, increasing disabling neurological conditions, increasing awareness of the atypical variants of BP, better diagnostic methods, and increased use of certain culprit drugs (1, 9). Growing evidence has shown that dipeptidyl peptidase IV inhibitors (DPP4i), used to treat diabetes mellitus, may be implicated in the development of BP (10–15). Findings from a recent systematic review and meta-analysis suggested that aldosterone antagonists, DPP4i, anticholinergics, and dopaminergic medications are associated with BP (16). Moreover, the number of BP cases associated with cancer immunotherapies using anti-programmed cell death protein 1 (anti-PD-1) and anti-programmed death ligand-1 (anti-PD-L1) has also increased impressively (17–21). Together, these findings highlight the importance of taking an accurate and detailed drug history for effective BP treatment.

Management of BP is often challenging. Currently, high-potency topical and/or systemic corticosteroids, at an initial dose of 0.5 mg/kg/day are used as first-line therapies (1, 22). In corticosteroid-dependent or relapsing BP, second-line treatments with conventional immunosuppressive drugs (e.g., methotrexate, azathioprine, mycophenolate) or safer immunomodulatory drugs (e.g., doxycycline, dapsone, omalizumab) may be considered. Immunosuppressants may reduce the required dosage and thus long-term side effects of corticosteroids; however, the use of these drugs is also limited by their side effects or efficacy. Regrettably, to date, there are no specific drugs that have been approved for the treatment of BP by the Food and Drug Administration. Indeed, even under the current mainstay of treatments, BP still carries a 2- to 3-fold higher mortality compared with the age- and sex-matched general population (1, 23–25). Advanced age, concomitant neurologic disease, poor general condition, and long-term use of high-dose corticosteroids are all risk factors for BP mortality in BP patients (23, 26, 27). The particular vulnerability of BP patients highlights the importance of identifying and developing an effective treatment capable of balancing safety and efficacy.

The diagnosis of BP is based on suggestive clinical features, positive direct immunofluorescence with linear deposits of IgG and/or complement C3 along dermal–epidermal junction and IgG against BP180 and/or BP230 by enzyme-linked immunosorbent assay (1). The pathogenic role of IgG autoantibodies in BP has been extensively studied. However, clinical presentation with intense pruritus and urticarial prodrome, laboratory findings with eosinophilia and elevated IgE, histopathology findings consist of sub-epidermal bullae with a dermal infiltrate of eosinophils and neutrophils, as well as recent expanding evidence from *in vitro* and *in vivo* experiments all implicate type II inflammation as an important driver of BP pathogenesis (28). Type 2 inflammation is the inflammatory pathway involving a subpopulation of CD4+ T cells that secrete IL-4, IL-5, and IL-13, B cells that secrete IgE, and granulocytes, such as eosinophils, mast cells, and basophils. These effectors in type II inflammation may serve as novel targets for BP treatments in future (29).

In this review, we consolidate existing and updated BP investigations that warrant a greater emphasis on the pathogenic role of type 2 inflammation in BP. We also discuss strategies for targeting type II inflammation as a treatment for BP and weigh the clinical efficacy of current and emerging targeted therapies.

## An updated understanding of the pathogenesis of bullous pemphigoid

Compared with pemphigus, which has a more straightforward pathogenic mechanism, as a B cell- and autoantibody-mediated autoimmune disease, the pathogenesis of BP appears to involve humoral autoimmunity against the antigen in the basement membrane zone as well as the activated inflammatory pathways (30). The upstream and downstream mechanisms implicated in blister formation in BP are the subject of many active investigations.

### Target antigens for autoantibodies in BP

BP is immunologically characterized by the presence of tissue-bound and circulating autoantibodies that target two hemidesmosomal proteins, BP antigen 180 (BP180, collagen XVII, or BPAG2) and BP antigen 230 (BP230 or BPAG1e) (2, 31–33). BP 180 is a 180-KDa transmembrane glycoprotein consisting of a globular cytoplasmic N-terminal domain, a short transmembrane stretch, and a large extracellular C-terminal domain containing 15 collagenous subdomains (COL1–COL15) interspersed by 16 non-collagenous sequences (NC1–NC16). BP180 contains multiple binding sites for hemidesmosome proteins, including the extracellular domains of integrin α6 and laminin-5 and the cytoplasmic domains of integrin β4, plectin, and BP230 (32, 34, 35). BP230 is an intracellular constituent of the hemidesmosomal plaque and belongs to a family of intermediate filament-binding plakin proteins. BP230 has a central rod domain flanked by globular end domains (35, 36).

The structure and location of BP180 indicate that it functions as a core anchor protein and thus plays a crucial role in the pathogenesis of BP. It is generally accepted that the immunodominant non-collagenous region 16A (NC16A) of the BP180 ectodomain is the main target for BP autoantibodies (37, 38). In a passive transfer animal model, antibodies against the ectodomain of BP180 were shown to be pathogenic, resulting in subepidermal vesiculation with C3 deposition at the basement membrane zone and infiltration of neutrophils in the upper dermis (39). In a later study, injection of human BP autoantibody into collagen XVII-humanized (COL17m–/–,h+) mice provided solid evidence of the pathogenicity of anti-BP180 NC16A autoantibodies (40). Moreover, studies have also shown that serum levels of autoantibodies to BP180 NC16A quantified by ELISA correlate with BP disease activity (41, 42). Higher anti-BP180 NC16A IgG titer at the time of treatment cessation is associated with a greater risk of relapse (43). However, it should not be ignored that some patients may have autoantibodies with reactivity to domains outside of NC16A, such as the C-terminal domain of BP180 and a 120-kDa fragment of BP180 known as linear IgA disease-1 (LAD-1). Most of these patients exhibit no erythema and display relatively milder phenotypes (44, 45).

Whereas the role of anti-BP180 antibodies has been extensively characterized, it was not until recently that the pathogenicity of anti-BP230 antibodies was documented in experimental BP models (36). Usually, IgG autoantibodies to BP180 are the first to be detected, followed subsequently by IgG autoantibodies to BP230 (44).

### Molecular mechanisms for blister formation in BP

The pathogenic mechanisms involved in blister formation after BP autoantibody binding are complex and under extensive investigation. Both complement-dependent and complement-independent pathways have been proposed. Proteolytic enzymes and cytokines orchestrating with the recruitment of inflammatory cells also participate in the process (2, 32).

A study by Liu et al. has shown passive transfer of rabbit antimurine IgG antibodies against BP180 can lead to blistering disease with key immunopathological features of BP via complement activation (46). Epitope mapping studies have demonstrated that pathogenic antibodies in this animal model recognize the murine BP180 NC14A region, which has sequence overlap with regions of the immunodominant human BP180 NC16A epitopes (47). Further studies using C4-deficient, C1q-deficient, mast cell-deficient, neutrophil-depleted, and neutrophil elastase-null [NE(-/-)] mice demonstrated that the mechanisms involved include complement activation, mast cell degranulation, neutrophil infiltration, secretion of proteases, and BP180 cleavage (46, 48–51). There are also clinical lines of evidence about complement-dependent pathogenic pathways. Deposition of complements at the basement membrane zone could be observed in the majority of skin biopsies from BP patients, and the detection of linear IgG and/or C3 deposits has been considered a diagnostic hallmark of BP (1, 3, 52).

However, *in vitro* and *in vivo* studies have also pointed toward the complement-independent pathways in BP pathogenesis. Through *in vitro* vibration assay, Iwata et al. demonstrated serum IgG from BP patients caused internalization of BP180 from the cell membrane, resulting in decreased cell adhesion to the bottom of the culture plate (53). Furthermore, in an *in vitro* model with C3-deficient BP180-humanized neonatal mice, Ujiie et al. showed that passive transfer of BP autoantibodies caused BP180 depletion in lesional skin without complement activation (54). Natsuga et al. demonstrated that passive transfer of F(ab')_2_ fragments of the human BP or rabbit IgG against BP180, of which the complement-binding Fc domains which were removed were still able to induce disease (55). Further evidence supporting complement-independent pathways was reported in a case study on two IgG4-positive BP patients without complement activation at the basement membrane zone (56). As we know that IgG4 does not bind complement effectively and is unable to activate the complement pathway, it is more likely that anti-BP180 IgG4 and IgE autoantibodies induced internalization of BP180 through macropinocytic mechanisms (57, 58). Importantly, the results from studies with complement knock-out mice, and F(ab')_2_ fragments should be carefully interpreted as these are not physiologic human conditions. Complement may not be absolutely indispensable but is still important in the amplification of inflammation and blister formation in BP. Complement-dependent and complement-independent mechanisms may coexist at the same time in the same patient (59).

### Pathogenic relevance of IgE autoantibodies in BP

Historically, most experimental studies investigating the pathogenesis of BP have focused on IgG class autoantibodies, despite an observed elevated total serum IgE level, a hallmark of type II inflammation, having been reported in 70–85% of BP patients (60). The presence of IgE autoantibodies in BP is particularly worthy of investigation in light of the fact that BP patients usually have an early urticarial phase, and the role of IgE-mediated degranulation of mast cells is well established in urticaria.

Like IgG, IgE autoantibodies in BP mainly target the BP180 NC16A domain, but regions outside the NC16A domain were also reported to have been recognized by IgE in some patients (61, 62). The percentage of BP patients with IgE anti-BP180 NC16A ranges from 22 to 100% according to different studies. This variation might be explained by the different testing platforms (ELISA or immunoblotting) employed and the use of diluted vs. undiluted serum (60). Importantly, the level of anti-BP180 NC16A IgE has been shown to correlate with BP disease activity (61, 63, 64).

In recent years, more laboratory studies have provided solid evidence that IgE-class autoantibodies are pathogenic in BP. Fairley et al. found that injecting purified anti-BP180 IgE from BP patients into human skin transplanted to athymic nude (nu/nu) mice induced erythematous elevated plaque, and histological separation of the epidermis from the dermis (at a higher dose of BP IgE) (65). Degranulation of mast cells and infiltration of eosinophils were also observed in the upper dermis during the process. Similar findings were shown in another animal model, where anti-BP180 IgE-producing hybridoma was injected subcutaneously in severe combined immunodeficiency (SCID) mice with engrafted human skin. At 21 days, test mice developed intense eosinophil infiltration and degranulation of mast cells within the grafts and histological basement membrane blisters (66).

Apart from FcRI-mediated mast cell degranulation by anti-BP180 IgE shown in the aforementioned animal model, and in circulating basophils/mast cells from BP patients (65–67), Messingham et al. suggested an FcR-independent mechanism for IgE class autoantibodies that could contribute to BP pathogenesis. Incubation of cultured human keratinocytes or human skin organ culture with IgE isolated from BP sera, or IgE class monoclonal antibodies against BP180 NC16A resulted in the release of IL-6 and IL-8 cytokines that may mediate disease development in BP. In the absence of immune inflammatory cells, IgE autoantibodies stimulated the production of IL-6 and IL-8 by keratinocytes and led to internalization and depletion of BP180 from the keratinocyte surface. The IgE Ab-mediated loss of hemidesmosomes might weaken the attachment of basal keratinocytes to the basement membrane zone, thereby contributing to blister formation (58, 68). Further studies are needed to explore the mechanism underlying anti-BP180 IgE-mediated tissue damage, and it will be particularly interesting to investigate the complex interplay between IgE and IgG class autoantibodies in BP pathogenesis.

### The role of eosinophils and Type II inflammation in BP

Type 2 inflammation is a pattern of immune response involving a subpopulation of CD4+ T cells known as Th2 cells that secrete IL-4, IL-5, and IL-13 and stimulate Type 2 immunity, which is characterized by high IgE antibody titers and eosinophilia. Several lines of evidence have pointed out a prominent type II inflammatory response in BP, such as the involvement of IgE (described in the section above), eosinophils, and Th2 cytokines and chemokines.

Eosinophil infiltration is a prominent histological feature in BP, and peripheral eosinophilia presents in around 50% of patients (69). Both increasing numbers of eosinophil in blood and tissue, as well as activation of eosinophil, have been shown in BP. The serum concentration of secondary granules, such as eosinophil cationic protein (ECP), is significantly elevated in BP patients and is decreased following remission after treatment (70). In line with this finding, Borrego et al. observed the deposition of eosinophil granule proteins in BP lesional skin (71). The deposition was seen in all stages, but most markedly in early erythematous urticarial lesions minimally in uninvolved skin.

Further evidence from experimental models has also supported the causal role of eosinophils in BP pathogenesis. In an *ex vivo* human model consisting of normal human skin cryosections activated with IL-5 and put in BP autoantibodies, eosinophils induced separation along the dermal-epidermal junction (DEJ) of *ex vivo* skin (72). Moreover, in a transgenic mouse model expressing human NC16A and human high-affinity IgE receptor (FcεRI), Lin et al. showed that anti-NC16A IgE from BP patients induced sub-epidermal split, eosinophil infiltration, and IgE deposition at the DEJ. Furthermore, IgE autoantibodies fail to induce BP in eosinophil-deficient mice, confirming that eosinophils are required for IgE-mediated tissue injury, and thus providing a further link between IgE autoantibodies, eosinophils, and blister formation, occurring independently of neutrophils (73).

Informed by eosinophil biology and emerging investigations on the role of eosinophils in BP, there are multiple potential mechanisms by which eosinophils may contribute to the pathogenesis of BP: (1) Eosinophils act as effector cells and exert direct action on dermal–epidermal separation, (2) Eosinophils produce cytokines and chemokines, which amplify and sustain a local immune response, and (3) Eosinophils play a role in BP-related pruritus (28).

The release of matrix metalloproteinase (MMP)-9, neutrophil elastase (NE), plasmin, and eosinophil cationic protein (ECP) by inflammatory cells can cleave and degrade BP180, thus leading to dermal–epidermal separation and blister formation (74, 75). Eosinophils have been shown to be the principal source of MMP-9 production in BP lesions (76). In another study conducted in Matrigel-coated chemotaxis chambers, MMP-9 was found to be involved in the transmigration of eosinophils through basement membrane components. In particular, the substrate-degrading activity of MMP-9 was increased in the presence of both IL-5 and platelet-activating factor (PAF) (77).

Autoreactive T-cell responses to BP180 in BP patients have been investigated. By testing *in vitro* responses of peripheral blood mononuclear cells from BP patients and healthy donors to BP 180, Büdinger et al. demonstrated that BP180-specific T-cell lines from BP patients produced both Th1 and Th2 cytokines whereas autoreactive T cells from control exclusively produced the Th1 cytokine (78). Similar results were observed in a recent study. Patients with BP showed a mixed Th1/Th2 response against BP180 while autoreactive Th1 cells were identified in a subset of elderly patients with pruritic disorders (79). Both findings strongly suggest that autoreactive Th2 responses to BP180 are restricted to BP patients and may thus be critical in the pathogenesis of BP.

The key cytokines and chemokines involved in eosinophil chemotaxis in BP are currently under active investigation. Increased levels of IL-4, IL-6, and IL-10 have been observed in the blister fluid of BP (80). Moreover, higher frequencies of skin-homing IL-4 and−13-producing T cells were found in BP skin lesions (81). The eosinophil chemoattractant IL-5, eotaxin, and receptor CCR3 were also found in high levels in blister fluid, lesional skin, and perilesional skin in BP. The levels of these markers correlate significantly with the number of dermal infiltrating eosinophils (82, 83). Following initial cytokine release, it is thought that intralesional eosinophils amplify a local type II inflammation by releasing additional cytokines and chemokines, such as eotaxin and human monocyte chemoattractant protein (MCP)-4 (84). As such, more eosinophils would be then recruited, resulting in a positive feedback loop formed. In theory, eosinophils will also express a series of cytokines that facilitate plasma cell survival and T-cell recruitment. B-cell-activating factors of the TNF family (BAFF) and activation and proliferation-induced ligand (APRIL) have been recognized as key regulators of autoimmune B-cell induction, both of which are expressed by eosinophils. BAFF and APRIL levels are significantly elevated in BP patients. Interestingly, BAFF and APRIL levels increase early in the development of the disease and thus appear to play a key role prior to the development of detectable levels of autoantibodies (85, 86). IL-16 and CCL5 [also known as regulated upon activation, normal T cell expressed and secreted (RANTES)] can induce migration of T lymphocytes. Eosinophils release both IL-16 and CCL5 (also known as RANTES) and thus can potentially amplify the immune response by the recruitment of CD4+ T cells. The precise effect of eosinophils on regulating and recruiting B cells and T cells in BP pathogenesis need to be further elucidated.

**Figure 1:**
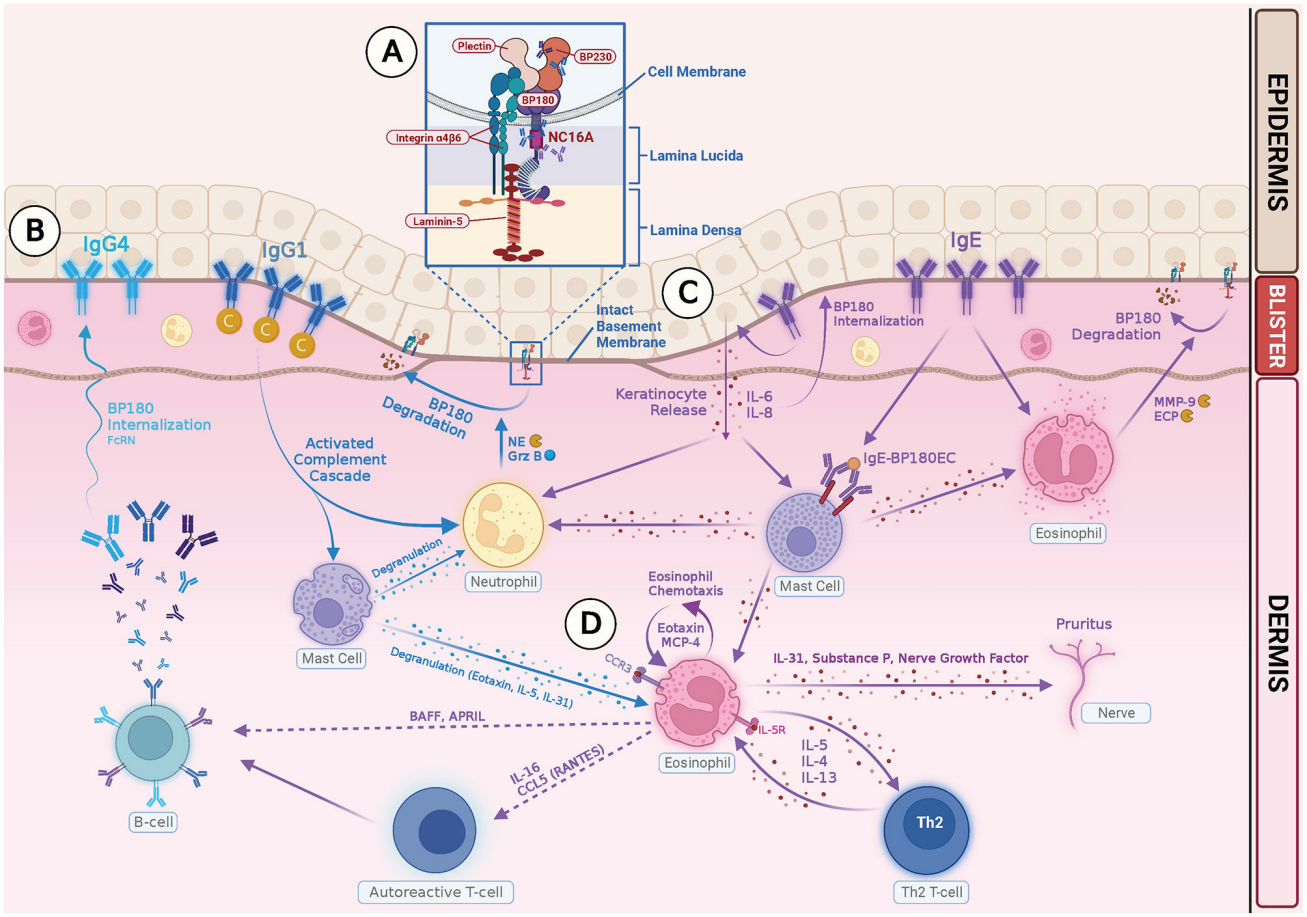
Illustration of IgG- and IgE-mediated pathogenesis of Bullous Pemphigoid. **(A)** Autoreactive T cells in BP patients stimulate B cells, producing pathogenic IgG and IgE autoantibodies. The NC16A ectodomain of transmembrane BP180 is the main target for both IgG and IgE autoantibodies. IgG reactivity with intracellular epitopes of BP230 has also been documented. **(B)** Binding of IgG1 class autoantibodies to the hemidesmosome leads to complement activation, mast cell degranulation, neutrophil and eosinophil infiltration, and degradation of BP 180 by releasing proteases, such as neutrophil elastase (NE) and granzyme B (GrzB). Binding of IgG4 class autoantibodies induces internalization of BP180 through complement-independent macropinocytic mechanisms. **(C)** Binding of anti-BP180 IgE to the hemidesmosome induces FcRI-mediated degranulation of mast cells and infiltration of eosinophils. Binding of IgE to the NC 16A domain also stimulates production of IL-6, IL-8 by keratinocytes, and internalization of BP180 from keratinocyte surface, which leads to reduced adhesion. **(D)** Eosinophils contribute to the pathogenesis of BP in several ways: (1) Eosinophils act as effector cells and exert direct action on dermal–epidermal separation by releasing MMP-9 and ECP. (2) Eosinophils produce Th2 cytokines and chemokines, which amplify and sustain a local immune response. (3) Eosinophils release IL-31 and play a role in pruritus.

Intensive pruritus is a characteristic feature of BP, and IL-31 is a known pruritogenic cytokine. In a recent study on the role of IL31 in BP patients, a high level of IL-31 expression was observed in BP blister fluids, and eosinophils were found to be a major source of IL-31 (87). Amber et al. proposed that eosinophils may play a role in BP-related pruritus through pathways involving IL-31, substance P, nerve growth factor, interaction with nerve cells, leading to enhanced growth and branching, and cross-talk with the autonomic nervous system (28). Figure 1 summarizes how IgG and IgE autoantibodies may be involved in the pathophysiology of BP.

There have been major advances in understanding the immunopathogenesis of BP through dissecting the role of IgE autoantibodies, eosinophils, and type II inflammatory cytokines. Understanding BP pathogenesis forms the basis for the identification of mechanism-based targeted therapies for BP patients.

## Repurposing biologics for the treatment of bullous pemphigoid

Because long-term steroid and immunosuppressant treatments are responsible for severe and even life-threatening side effects, it cannot be denied that there is a significant unmet need for safe and effective methods to treat bullous pemphigoid (22, 27, 88). Although there are still no US Food and Drug Administration (FDA) or European Medicine Agency (EMA)-approved biologics for BP, some biologics currently approved for other dermatologic diseases had been repurposed to treat resistant BP in recent years. The advantages of drug repurposing are the known safety profile of licensed drugs, and the decreased time and costs for drug approval.

### B-cell depletion therapy with Rituximab

Rituximab is a human–mouse chimeric monoclonal antibody directed against CD20, a transmembrane protein expressed by the B-cell lineage from the pre-B cell throughout the plasmablast stage. Binding of rituximab to CD20 leads to B-cell depletion and subsequently decreases antibody production (89). Rituximab is not only effective in treating B-cell lymphoma and chronic lymphocytic leukemia but also it has been licensed for the treatment of some autoimmune disorders, such as severe rheumatoid arthritis, granulomatosis with polyangiitis (GPA), and microscopic polyangiitis (MPA). Furthermore, based on promising results from a multicenter, randomized trial in France (Ritux3) (138), rituximab has been approved for the treatment of adults with moderate-to-severe pemphigus vulgaris by the FDA in 2018, followed by the EMA in 2019.

The demonstrated viability of treating pemphigus with rituximab leads to the inference that rituximab might also be beneficial to BP patients as well. In an earlier retrospective case–control study in Taiwan, comparisons between first-line combination therapy with rituximab plus corticosteroids and conventional therapy with corticosteroids only were studied. In the combination group, 92% (12 of 13) of patients achieved complete remission (CR), which is defined as a status of no established or new lesions for at least 2 months. Urinary tract infection and pneumonia were the leading infections observed in both groups, and there was no significant difference in the 1-year mortality rate in the two groups (90). In another retrospective review, 28 patients with pemphigoid diseases were treated with rituximab. Disease control (DC) was achieved in 67.9%, and partial remission (PR) or CR was achieved in 57.1% of the cases. IgA-dominant cases were found to have less DC, PR, and CR compared with IgG-dominant cases. During follow-up, 66.7% of patients relapsed but repeated treatment with rituximab led to PR or CR in 85.7% of the retreated cases. Importantly, among the three deaths reported, one death was possibly related to rituximab (91).

In a larger cohort study from the University of Pennsylvania, 38 patients were followed for at least 1 year after rituximab or until death. Overall, 29 of 38 patients (76%) achieved CR after a median of one rituximab cycle, and 39% achieved CR off therapy (CROT) after a median of two rituximab cycles. However, 17 of 29 patients (59%) relapsed a median of 6.2 months after achieving CR. Seven infectious serious adverse events were observed and there were two deaths, though they were deemed to be unrelated to rituximab (92). It is worth to note that the median anti-BP180 titer (available in 13 patients) significantly decreased 12 months after rituximab, but no significant differences were observed in anti-BP230 IgG titer, suggesting that anti-BP180 antibodies are predominantly produced by short-lived plasma cells, while anti-BP230 antibodies may be not. Interestingly, Hall et al. also found that after rituximab therapy for seven BP patients, there was a decrease in IgG anti-BP180 levels with a half-life of about 30 days, consistent with the half-life of IgG in the circulation. IgG anti-BP230 and IgE anti-BP180 levels did not decline as much, with persistent autoantibodies present during the follow-up period. The interpretation of these results might be that two populations of IgG autoantibodies exist, one arising from short-lived plasma cells associated with the pathogenesis of skin disease and a second group produced by long-lived plasma cells (93).

According to the above review and studies, rituximab is a therapeutic option in severe or recalcitrant BP. Although BP patients with rituximab treatment could achieve a significant reduction in disease activity, relapse and infectious adverse events are still major concerns. Careful and close monitoring for any signs of infection is crucial in BP patients, as older age, diabetes mellitus, renal impairment, azathioprine use, and corticosteroid equivalent to prednisolone >15 mg/day had been reported to be associated with an increased risk of infection during rituximab therapy (94). In the recent “updated S2 K guidelines for the management of BP initiated by the European Academy of Dermatology and Venereology,” rituximab was considered a third-line treatment in the most difficult-to-treat cases. Experts concluded the beneficial effects of using rituximab for treating BP are lower than pemphigus in terms of control of disease activity, clinical remission, relapse risk, potential severe side effects in debilitated elderly, and increased risk of COVID-19 infection (1).

### Biologics targeting type II inflammation

Omalizumab is a recombinant humanized anti-IgE monoclonal antibody. It blocks the binding of IgE to FcϵRI and FcϵRII on the surface of mast cells, basophils, and dendritic cells by specifically binding to the free IgE Cϵ3 region. The blockade then reduces the activation of inflammatory cells and the inflammatory cascade (95). Omalizumab has been licensed to treat moderate-to-severe persistent asthma, nasal polyps, and chronic spontaneous urticaria (CIU), and a wide range of conditions dependent on IgE triggering are being explored as being amenable to anti-IgE therapy (96).

The first case of a steroid-unresponsive BP patient who was successfully treated with omalizumab was reported in 2009 (97). The remarkable clinical improvement of this patient provided further support for the fact that IgE autoantibodies are relevant in autoimmunity. In this patient, eosinophil levels dropped during treatment; however, the measurement of total IgE may not have been accurate as the clearance rate of omalizumab:IgE complexes was slower and the complexes were not biologically active. In the study by Jafari et al., it was found that although omalizumab did not reduce total IgE serum levels in BP patients, levels of FcϵRI on circulating basophils decreased dramatically. Furthermore, a significant reduction of IgE+ and FcϵRI+ cells in BP lesional skin was observed in parallel with clinical improvement 4 weeks after treatment (98). These findings are in line with the theory that omalizumab causes dissociation of IgE from the IgE-FcϵRI complex, and IgE-free FcϵRI is unstable and would thus be internalized for degradation. Ultimately, the activation of mast cells and basophils will be reduced.

Since then, there have been more case reports showing the efficacy of omalizumab in BP. In real-world practice, many clinicians naturally think that omalizumab may particularly fit for BP patients with urticarial lesions and high serum IgE (1). In a recent case series, among 11 patients included, 54.5% experienced complete clearance of skin lesions after a median duration of 4.4 months on omalizumab. However, there was no significant difference in baseline IgE levels and eosinophil levels among full, partial, and non-responders (99). Similar results were concluded from a recent systemic review, in which 22 articles with 56 total BP patients were included. Due to the off-label use of omalizumab in the treatment of BP, optimal dosing has yet to be established. Most studies opted for the same regimen used in chronic idiopathic urticaria (300 mg SC every 4 weeks) and up-dosed as necessary. Overall, 87.5% of patients responded to treatment (55.4% CR and 32.1% PR) (100). There were no statistically significant differences in baseline eosinophilia and baseline IgE between the CR and PR/non-responders. These findings suggested that patient selection for omalizumab should not rely on IgE or eosinophil values. Another important takeaway from this systemic review was that disease flare-up occurred in half of the patients either due to omalizumab discontinuation or when systemic steroids were tapered; however, the response was recaptured with subsequent omalizumab administration in all patients for whom data were provided.

Omalizumab appears to be a promising treatment for BP with a good response rate and known excellent safety profile. It has been included in the “updated S2K guidelines for the management of BP initiated by the European Academy of Dermatology and Venereology” for second-line treatment (1). However, prospective, randomized controlled trials are still needed to determine whether omalizumab can be a monotherapy or an adjunctive agent to minimize exposure to broad-based immunosuppressants in BP patients. Moreover, identifying biomarkers to predict treatment response of omalizumab in BP patients is also an important direction for future studies.

Dupilumab is a fully humanized IgG4 monoclonal antibody that targets the alpha subunit of the interleukin-4 receptor (IL-4Rα), making it a receptor antagonist. Dupilumab binds to IL-4Rα and thereby inhibits IL-4 and IL-13 signal transduction, which are both considered Th2-signature cytokines (101). Dupilumab has been approved for the treatment of moderate-to-severe atopic dermatitis in adult and pediatric patients aged 6 months and older, as well as asthma, chronic rhinosinusitis with nasal polyposis, and eosinophilic esophagitis. Dupilumab has also demonstrated success in treating other pruritic or inflammatory skin diseases, such as prurigo nodularis, allergic contact dermatitis, and chronic hand eczema (102).

The development of dupilumab has not only led to a paradigm shift for the treatment of choice for atopic dermatitis but has also sparked research to better understand the role of type 2 inflammation in other skin conditions. It has been reported that increased numbers of interleukin-4- and interleukin-13-producing cells were found in both peripheral blood and blisters of bullous pemphigoid patients, and these numbers decreased after therapy with systemic corticosteroids (81). Based on this finding, in 2018, the first case report was published describing an elderly man with recalcitrant BP successfully treated with dupilumab. After 3 months of dupilumab monotherapy, the patient had complete resolution of all blisters, and undetectable levels of BP180 and BP230 antibodies (103).

Since this initial report, more case reports and case series have been published discussing the efficacy of dupilumab as a treatment for BP. In a multicenter case series, 92% (12 of 13) patients experienced disease clearance or satisfactory improvement with the desire to continue dupilumab. Total clearance of BP was achieved in 53.8% (7 of 13) of patients, and no adverse events were reported (104). Dupilumab was either used to lower the dose of systemic steroid or as a monotherapy. Some retrospective comparative studies have shown that the time to disease control, as well as the median time to reduce systemic steroid to minimal dosage were shorter in dupilumab groups compared to control groups. When the disease was controlled, the control dose and cumulative dosage of methylprednisolone in the dupilumab groups were lower than that of the control. Moreover, adverse events were also less frequent in the dupilumab groups (105, 106).

Takamura and Teraki reported a patient with BP who was successfully treated with dupilumab monotherapy (107). Treatment with dupilumab completely improved the pruritus within 2 weeks and skin blisters within 4 weeks. Biomarkers of BP such as blood eosinophilia, elevated serum levels of thymus and activation-regulated chemokine (TARC), and IgE also decreased, and presence of anti-BP180 antibodies became negative within just 7 months. In the circulation, the proportion of IL-4-, IL-13-, IL-17- and IL-31- producing CD4+ T cells significantly decreased after treatment, whereas the proportion of IL-4-, IL-13-, IL-17-, and IL-31-producing CD8+ T cells slightly decreased. These results suggest that BP is a Th2 disease, and that dupilumab exerts its therapeutic effect by suppressing effector Th2 cells in BP.

### Comparison of B-cell depletion therapy and type II inflammation targeting biologics

Although there still lacks direct head-to-head clinical trials comparing the efficacy and safety profile of rituximab, omalizumab, and dupilumab in BP, some insight can be gleaned from consolidating information from published systematic reviews and meta-analyses.

A recent systemic review included 75 studies with 211 BP patients receiving rituximab (*n* = 122), omalizumab (*n* = 53), and dupilumab (*n* = 36) treatment. Rituximab treatment led to complete remission in 70.5% and partial remission in 23.8% of patients within 5.7 months, with a recurrence rate of 20.5%. Omalizumab treatment led to complete remission in 67.9% and partial remission in 20.8% of patients within 6.6 months, with a recurrence rate of 5.7%. Dupilumab treatment led to complete remission in 66.7% and partial remission in 19.4% of patients within 4.5 months, with a recurrence rate of 5.6%. In rituximab group, 9.0% of patients expired and infection was the most common adverse event. Among patients who received omalizumab, one patient (1.9%) expired, and one case of thrombocytopenia was observed. There were no reported adverse events in the dupilumab group (108).

The recurrence rate in rituximab group seemed higher than that in omalizumab and dupilumab groups (20.5 vs. 5.7 vs. 5.6%), though this might be due to the longer disease duration of BP in rituximab group (25.4 vs. 9.6 vs. 19.2 months). In another systematic review published earlier in 2019, complete response rates were similar for rituximab and omalizumab groups (85 and 84%, respectively), with rituximab having a lower recurrence rate than omalizumab (29 vs. 80%) (109). More studies or controlled trials are needed to clarify this discrepancy regarding recurrence, as there are limitations in current systematic reviews, such as small sample size (particularly in omalizumab and dupilumab groups), and lack of standardization of baseline characteristics of patients and definition of clinical response across publications.

According to the available data, RTX only displayed a slightly higher remission rate, approximately 3% in CR and 3% in PR, than the other two biologics. Infectious adverse events and a certain percentage of deaths associated with RTX treatment were the major concerns. BP patients are often fragile because of their older age and multiple comorbidities; therefore, a treatment course balancing safety and efficacy is crucial. The final choice of which biologics may be most appropriate should depend on disease severity, previous response to treatment, patient's general condition, and presence of contraindications.

## Emerging therapeutic approaches targeting type II inflammation

The increasing incidence of BP in elderly people has created a strong demand for more specific, effective, and safe treatment options. In addition to repurposing existing biologics, a considerable number of clinical trials are underway pioneering new drugs, and many of them are specifically targeting the type II inflammatory pathway in the pathogenesis of BP.

### IL-5 inhibitors

Interleukin-5 (IL-5) is an important Th2 cytokine that plays a major role in eosinophil differentiation, proliferation, and recruitment. IL-5 is also reported to cause degranulation of eosinophils (110). Interleukin-5 was detected at a high level in BP blister fluid, and blister fluid IL-5 showed a significant direct correlation with ECP and the number of lesions in individual BP patients (82, 111). Moreover, IL-5 levels in active BP serum were much higher than in patients with clinical remission and in the healthy controls (112).

These findings suggest that IL-5 may be involved in the pathogenesis of blister formation and could be a therapeutic target in BP. However, there are limited published clinical data regarding IL-5-related therapies and present results are controversial. In a recent report, there was successful treatment response with intravenous reslizumab (an anti-IL-5 antibody) in a BP patient, resulting in rapid improvement of bullous lesions and allowing tapering of methylprednisolone dosage (113). Mepolizumab is a fully humanized monoclonal antibody that targets IL-5 and prevents binding to the IL-5 receptor on the surface of eosinophils. Mepolizumab has been approved for the treatment of asthma since 2015. However, in a randomized placebo-controlled, double-blind phase 2 pilot study for BP (NCT01705795), mepolizumab did not meet the primary endpoint set as the cumulative rate of relapse-free patients after initiating therapy (114, 115). A total of 30 patients were randomized in this study and received either 750 mg of mepolizumab or matching placebo every 4 weeks over 12 weeks in addition to prednisolone 0.5 mg/kg/day. It is possible this add-on therapy design may have obscured possible effects of mepolizumab, and the 12-week treatment period might have been too short to achieve long-term control of BP. An important observation is that although mepolizumab significantly decreased blood eosinophil numbers, it did not significantly affect tissue eosinophil infiltration in BP.

Benralizumab is a monoclonal antibody against the IL-5 receptor alpha subunit with therapeutic prospects in BP as it mediates antibody-dependent cell-mediated cytotoxicity of both eosinophils and basophils. A multinational, randomized, double-blind, phase 3 study to investigate the use of benralizumab as a treatment option for BP (FJORD) (NCT04612790) is currently in the recruitment stage. The proportion of participants who are in complete remission being off oral corticosteroids for more than 2 months at week 36 will be evaluated as the primary endpoints (116).

### Eotaxin and CCR3 inhibitors

In addition to IL-4 and IL-5, eotaxin, a chemokine, also plays a significant role in the recruitment of eosinophils into BP skin lesions (28, 84). Both eotaxin-1 (CCL11) and eotaxin-3 (CCL26) were found to be highly upregulated in the serum and blister fluid of BP patients and were associated with eosinophil numbers and activation (82, 117). Moreover, there was an increased expression of the specific eotaxin receptor, CC chemokine receptor 3 (CCR3) on eosinophils, and T cells infiltrating skin lesions in BP (83). The blockade of eotaxin or CC3R3 can provide potential novel treatment targets in BP.

Bertilimumab is a fully humanized monoclonal antibody that targets eotaxin-1 and has been evaluated in a completed phase 2, open-label clinical trial on nine patients with moderate-to-extensive BP (NCT02226146) (118). The study consisted of a treatment period with IV infusion of bertilimumab on Days 0, 14, and 28, and a follow-up period of approximately 13 weeks. The preliminary results demonstrated an 81% reduction in Bullous Pemphigoid Disease Area Index (BPDAI) scores and a steroid-sparing effect. The drug was well tolerated, and no significant adverse events were reported. Based on the result, FDA granted a fast-track designation to bertilimumab for the treatment of BP (119). Although promising, the efficacy of bertilimumab in BP should be further validated in randomized controlled trial with more patients and a longer follow-up duration.

AKST4290 is a potent and orally active CCR3 inhibitor and is expected to have anti-inflammatory and immune-modulating effects. There has been a completed randomized, double-blind, placebo-controlled clinical trial to assess the therapeutic effect and safety of adjunctive AKST4290 in subjects with mild-to-moderate BP (NCT04499235). Subjects received topical mometasone furoate concurrently with 400 mg AKST4290, or placebo, twice daily. The primary endpoint was the proportion of subjects who achieve disease control ( ≤ 3 new lesions/day and healing of existing lesions) without requiring rescue therapy. The study is completed, but the results are not yet available (120).

### IL-17 and IL-23 inhibitors

In patients with eosinophilia, the presence of IL-17 and IL-5 in combination act synergistically to enhance eosinophilic cytolytic activity (121). Indeed, it has been shown that innate immune cells produced IL-17 at BP lesional skin. Consistently, IL-17 upregulated the expression of MMP-9 and neutrophil elastase, the two proteases involved in blister formation (122). IL-23 is an important cytokine that promotes the expression of IL-17, thereby facilitating eosinophilia and contributing to inflammatory cell recruitment (123, 124). Based on these observations, targeting the IL-23/IL-17 axis may be another therapeutic option in BP.

In an animal model, IL-17A–deficient mice were protected against autoantibody induced BP, and pharmacological inhibition of IL-17A also prevented the induction of BP in mice (125). A case from real-world clinical experience, treatment with secukinumab, a humanized monoclonal antibody targeting interleukin IL-17, achieved a dramatic clinical improvement of both active BP and chronic psoriatic lesions (126). In another case report, secukinumab not only induced complete clearance of psoriatic skin lesions but also suppressed the serum anti-BP180-NC16a antibody level (127). The mechanism responsible for the decrease in autoantibodies after secukinumab treatment remains to be elucidated. Other than secukinumab, in a case reported that when a patient was treated with ixekizumab, another anti-IL-17A monoclonal antibody, psoriatic and BP lesions rapidly improved 2 weeks later, and both psoriasis and BP were still in remission after 1 year (128). Despite these promising results, however, an open-label phase 2 trial evaluating the activity of ixekizumab in BP (NCT03099538) failed to reach the primary endpoint—cessation of blisters following 12 weeks of therapy (129).

IL-23 inhibitors are also considered to hold potential in treating- BP. A phase 2 open-label, single-arm study (NCT04117932) is currently underway, and patients can still be recruited; as of current, 18 patients have been enrolled. The aim of the study was to evaluate the efficacy of ustekinumab, a monoclonal antibody targeting IL-12 and IL-23, in association with topical corticosteroids in BP patients during a period of 8 weeks. Complete remission from disease is the primary endpoint (130). An early open-label phase 1 pilot study to evaluate the effects of tildrakizumab, an anti-IL-23 antibody, in the treatment of BP has also been approved (NCT04465292) (131).

### Janus kinase inhibitors

Janus kinase inhibitors, commonly referred to as JAK inhibitors, are a novel small-molecule drug class that targets and blocks cytokine signaling mediated by the Janus kinase/signal transducer and activator of transcription (JAK-STAT) pathway, thereby regulating the immune response. Many chronic inflammatory skin diseases are associated with the release of proinflammatory cytokines, and the activation of intracellular JAK-STAT pathway. Therefore, JAK inhibitors become an attractive, targeted therapeutic approach in the dermatological field (132–134). Baricitinib, upadacitinib, and abrocitinib are JAK inhibitors that have been approved for the treatment of moderate-to-severe atopic dermatitis (AD), a disease driven by Th2 inflammation. JAK inhibitors abrogate the signaling of a wide range of proinflammatory cytokines in AD, including IL-4, IL-13, IL-22, IL-31, interferon gamma (IFN-γ), and thymic stromal lymphopoietin (TSLP). The simultaneous inhibition of multiple pathways contributes to superior efficacy and rapid improvement in both itch and skin lesions compared with biologics (135).

JAK inhibitors are anticipated to have therapeutic prospects in BP as pruritus and inflammatory skin lesions are both important features of BP, and there is increased expression of Th2 cytokines, such as IL-4, IL-5, IL-13, and IL-31, in BP patients. Moreover, JAK inhibition may be particularly beneficial when the pathogenesis of a disease is complex and mediated by multiple cytokines. Xiao et al. first reported a case of active BP concurrent with plaque psoriasis successfully treated with baricitinib. The patient showed complete remission of both bullous and psoriatic lesions without any adverse effects at the 24-week follow-up (136). In another case report, an elderly woman with BP and multimorbidity was successfully treated with upadacitinib at 15 mg daily. She had only partial response to systemic corticosteroid, and upadacitinib leads to complete resolution of disease and allowed tapering off prednisolone (137). Tofacitinib, a pan-JAK inhibitor, was also reported to be effective in severe BP, which was refractory to systemic steroid, mycophenolate, and even dupilumab and rituximab. Blisters and pruritus improved after instituting oral tofacitinib, with a relatively fast response of 3 weeks (138).

**Figure 2:**
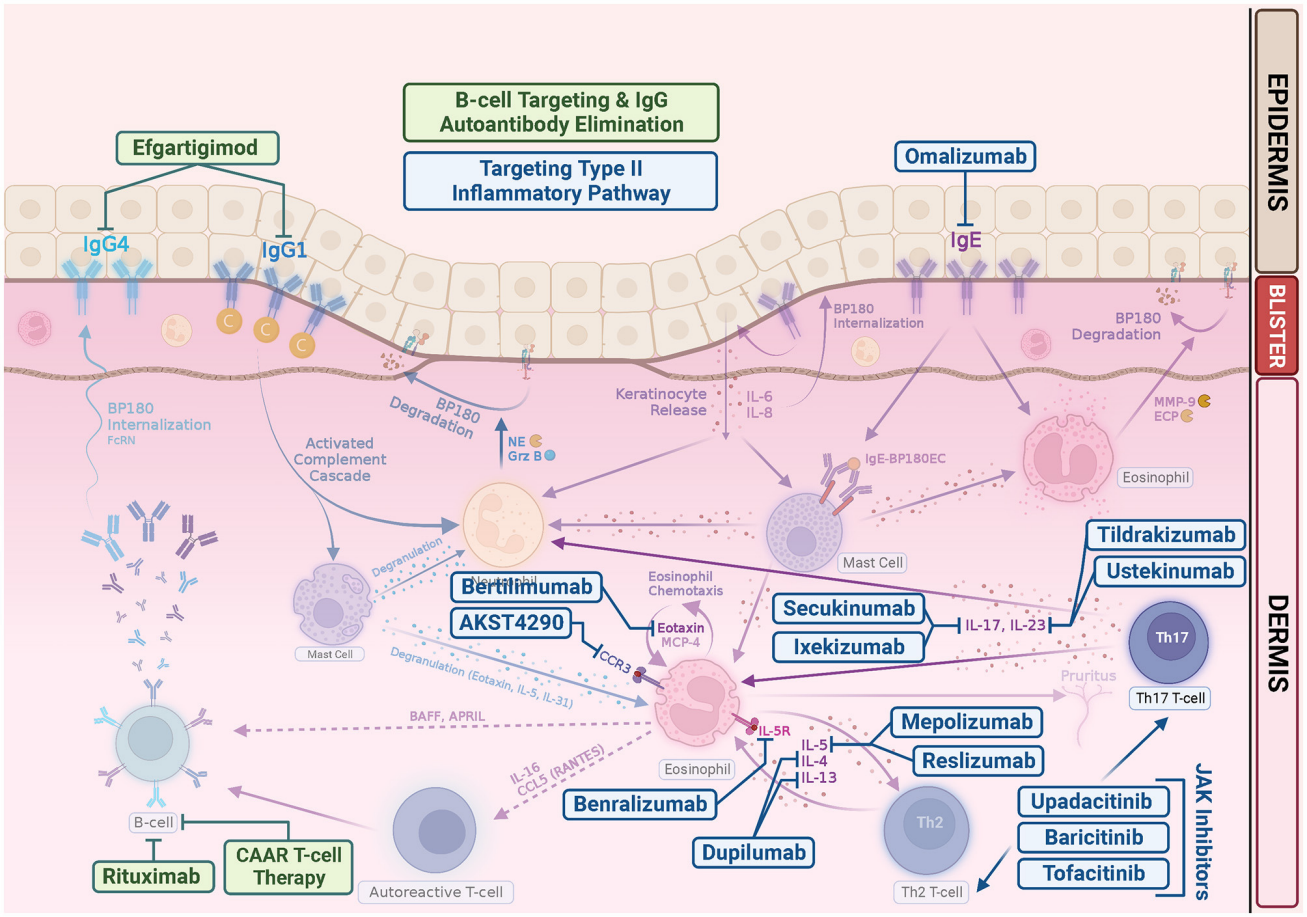
Emerging bullous pemphigoid therapeutic drugs and their targets. In addition to repurposing existing biologics, such as rituximab, omalizumab, and dupilumab, many clinical trials are underway pioneering new drugs for BP. Most target the Th2 inflammatory pathway in the pathogenesis of BP. Rituximab is a monoclonal antibody that targets CD20, which leads to B-cell depletion. Chimeric autoantibody receptor (CAAR) T cell may exhibit specific cytotoxicity and eliminate pathogenic B cells. Efgartigimod is a neonatal Fc Receptor (FcRn) antagonist, thus enhancing degradation of pathogenic IgG. Omalizumab is a monoclonal anti-IgE antibodies. Dupilumab is a monoclonal antibody that targets alpha subunit of IL-4 receptor, inhibiting both IL-4 and IL-13. Mepolizumab and reslizumab are monoclonal anti-IL-5 antibodies. Benralizumab is a monoclonal antibody against the IL-5 receptor alpha subunit. Bertilimumab is a monoclonal antibody that targets eotaxin-1, and AKST4290 is an orally active CCR3 inhibitor. Both ixekizumab and secukinumab target IL-17. Tildrakizumab is an anti-IL-23 antibody. Ustekinumab targets the p40 subunit of IL-12/23. JAK inhibitors can abrogate the signaling of a wide range of cytokines in BP, including IL-4, IL-5, IL-13, IL-17, and IL-31, suppressing Th2 inflammation.

To date, there is still a lack of large series or clinical trials on the use of JAK inhibitors in BP, although there is an ongoing randomized, double-blind, phase 2 study evaluating the treatment response of baricitinib for ocular inflammation in ocular mucous membrane pemphigoid (NCT05263505) (139). In future, it is likely that expanding studies will be conducted to investigate systemic and topical application of JAK inhibitor in the treatment of more autoimmune bullous diseases. Figure 2 is the schematic representation of current and emerging therapeutic drugs and their targets of bullous pemphigoid.

## Discussion

Bullous pemphigoid is one of the most common autoimmune bullous diseases. Treatment outcomes for BP with systemic corticosteroid and potent immunosuppressants are not satisfactory and have even been shown to be associated with significant morbidity and mortality. One of the primary reasons for BP's disease burden is that its patients are, on average, much older than patients with other autoimmune diseases. As such, the use of conventional immunosuppressive drugs, such as methotrexate, azathioprine, and mycophenolate are usually limited because of patient's multimorbidity and poor general condition, as well as possible drug interactions from polypharmacy and contraindications. Limitations regarding current BP treatments, as well as the increasing incidence of BP, highlight the medical need for pursuing novel therapies for BP patients. This unmet clinical need, as well as the increased knowledge of BP pathogenesis, will certainly accelerate the pace of translational research and clinical trials of new medications for BP.

Depleting B cells to induce long-term disease remission is a straightforward therapeutic approach for autoimmune bullous diseases. Seminal studies have validated that anti-CD 20 antibody therapy with rituximab is very effective for pemphigus, and it has been approved for the first-line treatment of moderate-to-severe pemphigus (140). Rituximab is also probably effective in BP; however, the evidence is not as convincing as that of pemphigus (1, 7, 93).(1) This difference in efficacy can be explained by the relative complexity of the pathogenesis of BP, which involves not only humoral autoimmunity against the autoantigens, but also activated inflammatory pathways. Moreover, the potential severe side effects in the debilitated elderly also remain a persistent concern. More specific targeted approaches for killing autoreactive B cells and depleting pathogenic IgG without general immunosuppression will be an important future research direction. Notably, a significant potential breakthrough has been achieved in the treatment of experimental pemphigus with the advent of chimeric autoantibody receptor (CAAR) T cells (141). Desmoglein (Dsg) 3 CAAR-T cells exhibit specific cytotoxicity against cells expressing anti-Dsg3 B cell receptors *in vitro* and specifically eliminate Dsg3-specific B cells *in vivo*. If effective for pemphigus, this therapy may also have the potential to treat bullous pemphigoid, in which the antigen is known and has been well characterized. There is another approach learning from the experience of treating other autoimmune disease. Neonatal Fc receptor (FcRn) has been shown to play an important role in the homeostasis of IgG. Efgartigimod is a FcRn antagonist that enhances degradation of pathogenic IgG. Efgartigimod first received approval for the treatment of generalized myasthenia gravis. Based on the effectiveness for other IgG-mediated autoimmune disease, a global phase 2/3, randomized, double-blinded, placebo-controlled study (BALLAD study) (NCT 05267600) to investigate the efficacy, safety, and tolerability of subcutaneous efgartigimod in BP is recruiting (142, 143).

There have been major advances in understanding the immunopathogenesis of BP through dissecting the role of IgE autoantibodies, eosinophils, and type II inflammatory cytokines. Omalizumab and dupilumab are the biologics targeting Th2 pathways that have recently been repurposed to treat resistant BP. The most attractive feature of targeting the Th2 axis for the treatment of BP is the superior safety profile (144). It can be imagined; therefore, omalizumab and dupilumab will be used much more frequently in treating BP during the pandemic and post COVID-19 era. Although these drugs are beneficial, additional work of well-designed controlled trials are needed to provide easier access to patients who cannot afford them as off-label prescriptions.

Targeting the Th2 axis and inhibiting the downstream inflammatory pathways is considered a promising strategy for developing novel BP treatments. Monoclonal antibodies against eotaxin, CCR3, IL-5, IL-17, and IL-23 are now tested in clinical trials. Small-molecule JAK inhibitors target and block cytokine signaling, and increasing evidence has suggested that JAK inhibitors can treat variety of inflammatory dermatological diseases. The use of JAK inhibitors for BP is also of great interest. Further investigation is needed to not only better understand the effectiveness but also illustrate the safety profile of JAK inhibitors in BP.

Having more targeted therapeutic choices for BP patients will be beneficial. These mechanism-based, targeted therapies hold promise to offer a safer and more effective alternative to our patients and is expected to be available for the treatment of BP soon. Optimized dosing regimen and the development of combination therapy and even tailored treatment that will lead to long lasting remission can also be anticipated further down the line.

## Author contributions

WT and H-EL contributed equally to conceptualization and the writing of the original draft. All authors contributed to manuscript revision and approved the submitted version.
